# Short-term outcomes following open gluteus maximus transfer for the management of hip abductor tears

**DOI:** 10.1093/jhps/hnad014

**Published:** 2023-06-10

**Authors:** Nicholas J Lemme, Myles Dworkin, Patrick J Morrissey, Edward J Testa, Daniel Kwan, Lauren Roussel, Ramin Tabaddor

**Affiliations:** Department of Orthopedic Surgery, Warren Alpert Medical School of Brown University, 2 Dudley Street, Providence, RI 02906, United States; Department of Orthopedic Surgery, Warren Alpert Medical School of Brown University, 2 Dudley Street, Providence, RI 02906, United States; Department of Orthopedic Surgery, Warren Alpert Medical School of Brown University, 2 Dudley Street, Providence, RI 02906, United States; Department of Orthopedic Surgery, Warren Alpert Medical School of Brown University, 2 Dudley Street, Providence, RI 02906, United States; Department of Plastic Surgery, Warren Alpert Medical School of Brown University, 593 Eddy Street, Providence, RI 02912, United States; Department of Plastic Surgery, Warren Alpert Medical School of Brown University, 593 Eddy Street, Providence, RI 02912, United States; Department of Orthopedic Surgery, Warren Alpert Medical School of Brown University, 2 Dudley Street, Providence, RI 02906, United States

## Abstract

Tears of the gluteus medius and minimus are an important cause of recalcitrant greater trochanteric pain syndrome. Although endoscopic and open abductor repairs have demonstrated promising outcomes, the success of these techniques is dependent on the size of the tear and the quality of the tissue. In patients presenting with abductor insufficiency and evidence of previous repair failure, large retracted tears, muscle atrophy and/or fatty infiltration, reconstruction/augmentation techniques should be considered. In the present study, we present a retrospective cohort study assessing patient outcomes following open gluteus maximus transfer for irreparable or severely retracted gluteus medius tears. Patients were included in the present study if they underwent open gluteus maximus transfer to address hip abductor tears that a senior surgeon deemed irreparable or at high risk for failure following isolated repair secondary to the following tear characteristics: large tears with >2 cm of retraction, the presence of extensive fatty infiltration (Goutallier Grade 3 or greater) and/or patients requiring revision abductor repair due to primary repair failure with associated pain and a Trendelenburg gait. Patients undergoing a concomitant, or those with a previous history of hip arthroplasty, were excluded from the study. All participants were prospectively enrolled in the study, and both pre- and post-operative patient-reported outcomes were collected at 6 months and 1 year including the modified Hip Harris Score, Visual Analog Score, Hip Outcomes Score of Activities Daily Living, Hip Outcomes Score for Sports-Related Activities and Overall Satisfaction with Hip. Pre-operative scores were compared with post-operative assessments using Student’s t-test with a significance level of *P* < 0.05. Twenty-one patients and 22 hips were included. The average age was 69 (SD ±9.2) and 17 (81%) were females. The average body mass index was 30.0 (±6.2). The outcome scores at both 6 months and 1 year demonstrated significant improvements compared with pre-operative functional assessment. This article reports the utility of gluteus medius/minimus repair augmentation or reconstruction via gluteus maximus transfer demonstrating improvement in patient-reported outcomes at short-term follow-up.

## INTRODUCTION

Tears of the gluteus medius and minimus tendons, commonly referred to as the hip abductors, are a significant cause of recalcitrant lateral hip pain and one of the most common causes of ‘greater trochanteric pain syndrome’. Such pathology predominately affects 50- to 70-year-old adults and is more commonly observed in females, compared with male patients [1]. Patients present with complaints of dull pain localized to the lateral hip which is exacerbated with weight-bearing, pain with palpation of the greater trochanter and a positive Trendelenburg gait [1]. The onset of symptoms is often insidious which may lead to a delay in diagnosis and treatment prior to being confirmed with magnetic resonance imaging (MRI). These patients are initially managed conservatively with a combination of oral anti-inflammatories, corticosteroid injections, shock-wave therapy and physical therapy. Outcomes, however, are variable with success rates ranging from 50% to 90% [2–4]. Surgical intervention should be considered for patients with persistent pain and abductor weakness despite conservative treatment [5].

Surgical management includes direct repair of the hip abductors utilizing either an open or endoscopic approach. In a recent meta-analysis of open and endoscopic abductor repairs, Looney et al. demonstrate good or excellent outcomes in 75% of patients, which suggests that one-fourth of patients undergoing isolated repair continue to have pain and disability [6]. In a recent systematic review, Chandrasekaran et al. demonstrated that patient success following primary abductor repair is highly dependent on the characteristics of the tear such as the tear size, degree of retraction, tissue quality and presence of fatty infiltration [7].

Various techniques have been described to address irreparable abductor tears and tears with advanced degeneration and/or fatty atrophy which may compromise results following isolated primary repair [8–10]. Techniques include Achilles allograft augmentation, human dermal allograft augmentation, vastus lateralis advancement and gluteus maximus transfer [8–10]. To date, studies investigating gluteus maximus transfer to augment primary repair or address irreparable hip abductor tears are sparse [6–8]. The current study aimed to perform a retrospective review of prospectively collected patient-reported outcomes following gluteus maximus transfer for the treatment of irreparable tears, revision tears with poor tissue quality or abductor tears with high-grade fatty infiltration. The authors hypothesized that following gluteus maximus transfer, patients would demonstrate improvements in hip abduction strength, pain and patient-reported outcome measures.

## METHODS

### Patient selection

Data for all patients who underwent a hip preservation procedure with the senior surgeon (R.T.) between 2018 and 2020 were prospectively enrolled into the study and retrospectively reviewed. Patients who failed at least 6 months of conservative treatment—which included anti-inflammatory medications, corticosteroid injections and/or physical therapy—and presented with persistent lateral hip pain, abductor weakness and a Trendelenburg gait were deemed candidates for a gluteus maximus transfer by the senior surgeon if they met the following criteria: (I) had an MRI demonstrating a gluteus medius (± minimus) tear that the senior surgeon deemed irreparable or at high risk for failure following isolated repair secondary to the tear characteristics [tear retraction >2 cm and/or extensive fatty infiltration (Goutallier 3 or greater)] and (ii) patients requiring revision abductor repair due to primary repair failure with associated pain and a Trendelenburg gait. Degree of fatty infiltration was evaluated and classified using the Goutallier/Fuchs classification system [11]. Of note at the time of surgery, if the gluteus medius/minimus tendons were able to be advanced to the native insertion sites without excessive tension, they were also repaired and incorporated into the gluteus maximus flap.

Patients who underwent a gluteus maximus transfer for abductor insufficiency were considered for inclusion in the study if they met all of the following criteria: (i) >18 years old, (ii) English speaking, (iii) had a minimum follow-up of at least 1 year, (iv) completed all pre- and post-operative patient-reported outcomes. Patients undergoing a concomitant, or those with a previous history of hip arthroplasty, were excluded from the study. Radiographic evaluation of each patient was performed using the Tönnis classification system [12]. Patients with Tönnis grade 3 degenerative changes were excluded from the study.

### Surgical technique

**Fig. 1.**
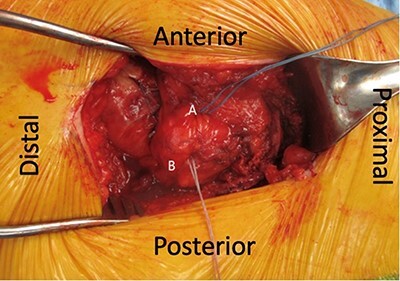
Left hip. Placement of bone anchors into greater trochanter. Anchor placed in the anterior facet of the greater trochanter (A). Anchor placed in the mid aspect of the lateral facet of the greater trochanter (B).

All of the gluteus maximus transfers were performed by the senior surgeons, R.T. (board-certified, fellowship-trained, CAQ-certified sports and medicine orthopedic surgeon) and D.K. (board-certified plastic and reconstructive surgeon). Patients were positioned on the operative table in the lateral decubitus position using a beanbag. A 6 cm mid-line incision was made centered along the tip of the greater trochanter. Blunt dissection was performed to identify the iliotibial band (ITB) and gluteus maximus. Dissection to identify the anterior edge of the gluteus maximus muscle and the posterior edge of the tensor fascia latae (TFL) muscle was performed. Once the ITB was exposed, an incision was made in line with the ITB. A trochanteric bursectomy was performed, and adhesions between the ITB and the abductors/vastus lateralis were bluntly taken down with finger dissection and subsequently excised with Metzenbaum scissors. The vastus lateralis and gluteus medius/minimus were inspected and probed to identify any defects. The fibrous tissue at the tear sites was resected, and the gluteus medius and minimus muscle tendon complexes were mobilized by freeing up the sub-gluteal space and the intergluteal space between the gluteus maximus and the gluteus medius using blunt finger dissection. Soft tissue was debrided off the greater trochanter using a Rongeur to identify and prepare the appropriate tendon footprints. Upon successful release, the authors determined whether the abductor muscle tendon complexes were able to be advanced to their native insertion sites without excessive tension and be repaired or not. If it was deemed reparable, next, two single-loaded 4.5 mm Polyetheretherketone (PEEK) Healicoil anchor (Smith & Nephew, London, UK) were placed in the anterior and mid-lateral facets, respectively. The anchor sutures were passed through the gluteus medius and minimus tendons with a free needle in horizontal mattress fashion and tied down. The repair was then probed to ensure stability and appropriate tension. The ends of the tied sutures were snapped and kept in place to be later incorporated into the gluteus maximus flap (Fig. 1). If the gluteus medius was deemed unrepairable, then a gluteus maximus flap was performed in isolation.

**Fig. 2.**
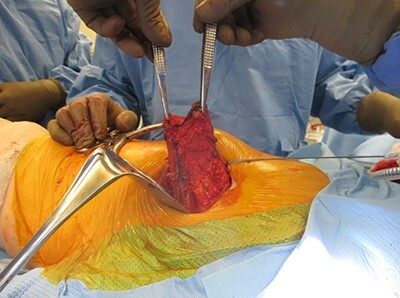
Left hip. Harvest and elevation of gluteus maximus myofascial flap. Left of screen, caudal. Top of screen, anterior.

The gluteus maximus flap was then planned and developed by the plastic surgery team, including the senior surgeon D.K. A triangular flap was created which included the anterior one-third of the gluteus maximus and posterior one-third of the tensor fascia (Fig. 2).

**Fig. 3.**
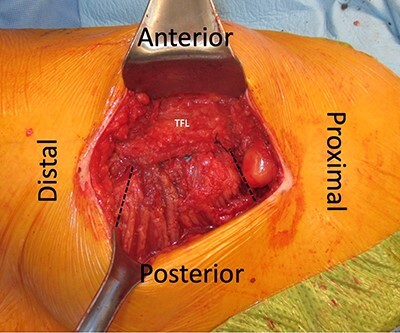
Left hip. Dotted lines demonstrating gluteus maximus flap secured in place deep to the TFL.

Once an adequate flap was developed, the flap was then positioned appropriately over the gluteus medius and minimus repair, and tensioning was tested by taking hip through range of motion and holding the gluteus maximus flap with Allis clamps in an appropriately tensioned position along the vastus ridge. Next, 2 × 4.5 mm PEEK Healicoil anchors were placed, one along the anterior aspect of the vastus ridge and the other along the posterior aspect of the vastus ridge. Both anchors were double loaded and passed through the gluteus maximus flap from anterior to posterior in a horizontal mattress fashion using a free needle. The sutures were then secured down. One limb of each suture was then fixed to a distal row using a 4.5 mm PEEK footprint anchor placed just distal to the central aspect of the vastus ridge. The suture limbs of the tied sutures of the gluteus medius and minimus repairs were then passed through the anterior border of the gluteus maximus flap in horizontal mattress fashion and secured down. This further secured and fixated the flap (Fig. 3). The hip was then taken through a full range of motion to ensure appropriate tension.

A complex closure was then performed by a plastic surgery team. This included 10 cm of undermining performed along the abductor fascial plane. A 15 Blake drain was then placed in this plane exiting the anterior hip and secured with a 3-0 nylon suture. Following this, the IT fascia was approximated with a figure-of-8 #0 Polydioxanone (PDS) sutures distally. Proximally, the remaining fascia was approximated as a flap over the gluteus maximus muscle with a figure-of-8 #0 PDS suture. The subcutaneous fascia was approximated with interrupted 2-0 PDS sutures. The dermis was approximated with buried interrupted 3-0 PDS sutures. The epidermis was approximated with running sub-cuticular 4-0 PDS. After cleaning the skin, a dry sterile dressing was placed upon the closed wound.

### Rehabilitation

Post-operatively, patients underwent a standardized post-operative protocol with an expected completion time being 6 months following the index procedure. Patients were made touch-down flat foot weight-bearing for 6 weeks. Abduction exercises against gravity and gait training were gradually implemented at 6 weeks following the index procedure with a licensed physical therapist. All patients received aspirin 325 mg twice a day for 6 weeks for prevention of deep venous thrombosis.

### Outcome scores and statistical analysis

All patients completed surveys prior to surgery as well as at 6 months and 1 year following their procedure. The patient-reported outcomes (PROM) assessed were the modified Harris Hip Score (mHHS), Visual Analog Pain Scale (VAS), Hip Outcome Score for Activities of Daily Living (HOS-ADL) and Hip Outcome Score Sport-Specific Subscale (HOS-SSS).VAS was assessed for patients at rest, with daily activities and with athletic activity [13]. Pre- and post-operative hip abductor strength was assessed via manual muscle testing. The minimal clinically important difference (MCID) for each PROM and time point was calculated using the distribution-based method originally described by Norman et al. [14]. The MCID was equal to one-half of the standard deviation of the delta in PROMs between time point 0 and 6 or 12 months.

All statistical analysis was performed using SPSS-25. Descriptive statistics were provided for demographic variables. Pre- and post-operative strength and outcome scores were compared using Student’s t-test. Statistical significance was set at *P* < 0.05.

## RESULTS

There were 21 patients and 22 hips that underwent hip abductor repair with gluteus maximus transfer. The average age was 69 (SD ±9.2), and 17 (81%) were females. The average body mass index (BMI) was 30.0 ± 6.2. Of these patients, 4 (19%) underwent isolated gluteus maximus transfer as a revision for previous failed open abductor repair. The mean Goutallier grade on pre-operative MRIs was 3.2 (SD 0.81). The mean Tönnis grade was 1.3 (SD 0.57). The mean hip abduction strength evaluated with manual muscle testing pre-operatively was 2.3 (SD 0.58). Post-operatively, hip abduction strength significantly improved at both 6- and 12-month follow-up (Table I). A statistically significant improvement in strength was also observed between the 6- and 12-month follow-up periods (Table I). All scores apart from the HOS-SSS demonstrated significant improvements at both 6 months and 1 year following the procedure (Table I). In regard to the HOS-SSS, our data demonstrated no significant improvement at 6 months; however, the observed improvements became statistically significant at 1 year (Table I). The MCID values at 6 months and 1 year, respectively, were as follows: mHHS (7.0 and 9.0), VAS (3.4 and 2.9), HOS-ADL (18.4 and 18.5), Hip Outcomes Score for Sports-Related Activities (HOS-SRA) (19.2 and 20), overall satisfaction (1.4 and 1.6) and abduction strength (0.45 and 0.40). The percentage of patients who achieved MCID at 6 months and 1 year time points in each PROM was as follows: mHHS (88% and 83%), VAS (91% and 100%), HOS-ADL (71% and 83%), HOS-SRA (67% and 70%), overall satisfaction (92% and 85%) and abduction strength (82% and 91%). Apart from strength, no significant difference was observed for any scores between the 6 month and 1-year follow-up period.

**Table I.** Patient-reported outcome scores pre- and post-operatively hip abductor repair with gluteus maximus transfer

	*Pre-operative scores* *average (SD)*	*6 months* *average (SD)*	*P-value*	*1 year* *average (SD)*	*P-value*
mHHS (max score 91)	30.3 (±10.3)	56.7 (±20.8)	<0.01	51.6 (±26.2)	<0.01
VAS (max score 30)	18.2 (±6.9)	7.9 (±6.6)	<0.01	11.7 (±9.0)	<0.05
HOS-ADL (percentage out of 100)	25.7 (±18)	68.6 (±22.8)	<0.01	59.6 (±27.5)	<0.01
HOS-SRA (percentage out of 100	8.8 (±19)	21.3 (±34.8)	0.27	29.8 (±36.9)	0.07
Overall Satisfaction with Hip (max score 10)	0.6 (±1.3)	7.5 (±2.4)	<0.01	4.8 (±3.9)	<0.01
Hip abduction strength (max score 5)	2.3 (±0.6)	3.9 (±0.7)	<0.01	4.2 (±0.6)	<0.01

## COMPLICATIONS

There was one patient who developed a deep infection of a seroma which required irrigation and debridement. No deep venous thrombosis or re-tears were identified.

## DISCUSSION

The current study presents a novel surgical technique using a mini-open approach to augment or reconstruct hip abductor tendon repairs which have failed prior fixation or were at risk of primary failure due to tissue degeneration and high levels of fatty infiltration (Goutallier 3 or greater). Using this technique, we demonstrated significant improvements in patients’ functional status and a reduction in pain, as demonstrated by meaningful improvements in post-operative patient-reported outcomes such as the mHHS, VAS and HOS-ADL.

Greater trochanteric pain syndrome can be extremely debilitating, affecting quality of life and functional status. Historically, it was believed that the primary cause of recalcitrant lateral hip pain was trochanteric bursitis, however, over the past decade, it has become clear that many of these patients suffer from tears to their gluteus medius and/or gluteus minimus tendons. Primary hip abductor repair has been demonstrated to be effective for partial tears or acute tears without significant retraction or degeneration [5, 15]. In a recent meta-analysis which included 22 studies and 611 hips, Longstaffe et al. demonstrated a low overall re-tear rate of 3.8%, however it is important to note that 78% of the tears included in the study were only partial thickness. Higher failure rates and inferior patient-reported outcomes following primary abductor repair have been associated with increased muscle atrophy and fatty infiltration commonly associated with more chronic tears. Similar to rotator cuff tears, the degree of atrophy and fatty infiltration can be graded using the Goutallier classification system [16]. Bogunovic et al. demonstrated a 29% failure rate following endoscopic abductor repair in patients with Grade 3 or 4 fatty infiltration compared with a 0% failure rate in patients with Grade 1 or 2 tears [11]. Similarly, Makridis et al. demonstrated that patients with significant hip abductor atrophy undergoing primary abductor repair had significantly higher levels of persistent pain and worse patient-reported outcomes post-operatively. Furthermore, 21% of patients with evidence of muscle atrophy reported persistent significant disability post-operatively compared with only 2.5% in patients with no evidence of atrophy [16]. These findings demonstrate the importance of diagnosing hip abductor tears early in order to minimize the proportion of patients undergoing surgical repair with significant atrophy or fatty infiltration. Furthermore, patients who present with long-standing hip pain and evidence of atrophy and/or fatty infiltration on MRI should be properly counseled regarding the increased risk of re-tear, clinical failure and the possible need for a gluteus maximus flap transfer.

There is currently no gold standard for repair of hip abductor tears. Techniques that have been described to date include repair augmentation with acellular dermal matrix, reconstruction with Achilles allograft and various isolated or combined tendon transfer techniques involving the gluteus maximus, TFL or vastus lateralis [9, 10, 17–20]. Tendon transfer and augmentation have multiple benefits. They have been shown to be cost-effective compared with allograft reconstruction or dermal matrix augmentation and may restore anatomic hip biomechanics as the transferred tendons reproduce the line of force produced by the deficient muscle with minimal donor site morbidity. Whiteside first described combined transfer of the gluteus maximus and TFL to address complete abductor insufficiency in patients with a history of total hip arthroplasty using two flaps from the gluteus maximus to recreate the short external rotators and hip abductors, respectively [10]. Of the 11 patients included, nine patients were able to achieve strong hip abduction and a negative Trendelenburg gait at follow-up. The study, however, was limited due to the lack of established patient-reported outcome measures [10].

Since this time, multiple studies have been performed investigating the utility and outcomes of gluteus maximus transfer for hip abductor insufficiency in native hips. Chandrasekaran et al.’s was one of the first studies to report their results following three patients who underwent a combined transfer of the gluteus maximus and TFL for primary hip abductor insufficiency. Despite the small sample size, they demonstrated that all three patients to have improvement in all of the studied PROs and abductor strength at a 1-year follow-up time [21]. Maldonado et al. subsequently further investigated this technique on a larger patient population which included 18 patients with a minimum of 1-year follow-up [18]. Following combined transfer of the gluteus maximus and TFL, the authors reported improvements in the hip abductor strength in 41% of their patients and a mean improvement of 25.2 points for the mHHS, which reached statistical significance, and 19.2 points on HOS-SSS, which did not reach statistical significance [18]. Similar improvements were observed in the present study with our patients demonstrating a mean improvement of 21.3 points for the mHHS and 21.0 points for the HOS-SSS score. Like Maldonado et al., while statistical significance was achieved for the improvements in the mHHS, the HOS-SSS scores did not reach statistical significance. Additionally, the patients in the present study reported their overall satisfaction with procedure to be 7.5 and 4.8 out of 10 at their 6-month and 1-year follow-up, respectively. The likely explanation for the lack of statistically significant improvement observed in either study for the HOS-SSS score is that this metric compares post-operative sports-related activity to pre-procedural levels and scored as a percentage of pre-injury activity. As a result, it may not be the most appropriate outcome measurement for patients typically undergoing this surgical intervention, as they are generally elderly (mean age, 69 years), lower in demand and less likely to participate in sporting activities.

The current study demonstrates that a gluteus maximus transfer for augmentation of abductor repairs or reconstruction for irreparable abductor tears can be performed to achieve acute pain relief as well as promote longer-term clinical benefits. Our ability to demonstrate similar outcomes as Maldonado et al. indicate that gluteus maximus transfers can reliably produce good clinical results. Additionally, in contrast to the study by Maldonado et al., the present study included patients undergoing gluteus maximus transfer following a failed primary repair, which indicates that the gluteus maximus transfer remains an acceptable technique even in the revision setting. Furthermore, we describe a technique using a mini-open approach. The smaller incision provided sufficient visualization of pathology without excessive violation of surrounding tissue which results in improved cosmetics and reduced recovery time [11].

The strengths of the current study included (i) the inclusion of patients whom only underwent isolated abductor repair without any concomitant intra-articular interventions and (ii) the ability to collect validated patient-reported outcomes on all of our patients. Despite this, several limitations do exist. First, this study was a retrospective study and no control group was used in the study to compare the outcomes of the present cohort to a non-operative cohort. Second, only patients who completed all questions during follow-up were included in the study. This resulted in attrition and a variable number of patients included at both 6-month and 1-year follow-up. As a result, our findings demonstrate a large standard of deviation in several patient-reported outcome measures (PROM) and may have exposed the study to selection bias. Third, in the present study, the data were prospectively collected and retrospectively reviewed, and we did not perform a formal gait evaluation pre- or post-operatively. Therefore, we were not able to provide data regarding Trendelenburg gait and/or improvements in gait following surgical intervention. Additionally, we had inconsistent documentation regarding whether patients had improvement in their trochanteric pain following surgery. However, the PROs used in the present study may act as a surrogate for the data points. For example, the mHHS score includes questions regarding pain, gait function and ability to perform functional activities. Finally, post-operative MRI’s were not performed; therefore, we were unable to provide data related to post-operative healing in the present study. Finally, the present study only includes patient-reported outcomes up to a 1-year follow-up time, and therefore, we were unable to determine whether sustained improvements were observed at a longer follow-up time.

In summary, the present study demonstrates the utility of the gluteus maximus transfer technique for augmentation of hip abductor repairs and reconstruction for irreparable tears in patients with chronic large, retracted tears with associated fatty infiltration or repair failures, which would otherwise put the patients at high risk for failure in the setting of isolated primary repair. We demonstrated statistically significant improvements in all PROMs at both 6 months and 1 year of follow-up with no patients demonstrating evidence of clinical failure nor re-tear at minimum follow-up time of 1 year. Additional studies are needed to further evaluate whether the observed benefits can be sustained at later time points. It is prudent that this technique be compared with other augmentation and reconstruction techniques to determine the gold standard of care for hip abductor tears which are not amenable to isolated primary repair.

## Data Availability

The data that support the findings of this study are available on request from the corresponding author, N.J.L. The data are not publicly available due to restrictions, e.g. their containing information that could compromise the privacy of research participants.

## References

[1] Lindner D, Shohat N, Botser I et al. Clinical presentation and imaging results of patients with symptomatic gluteus medius tears. *J Hip Preserv Surg* 2015; 2: 310–5. 27011854 10.1093/jhps/hnv035 PMC4765298

[2] Torres A, Fernández-Fairen M, Sueiro-Fernández J. Greater trochanteric pain syndrome and gluteus medius and minimus tendinosis: nonsurgical treatment. *Pain Manag* 2018; 8: 45–55. 29182042 10.2217/pmt-2017-0033

[3] Mautner K, Colberg RE, Malanga G et al. Outcomes after ultrasound-guided platelet-rich plasma injections for chronic tendinopathy: a multicenter, retrospective review. *PM R* 2013; 5: 169–75. 23399297 10.1016/j.pmrj.2012.12.010

[4] Rompe JD, Segal NA, Cacchio A et al. Home training, local corticosteroid injection, or radial shock wave therapy for greater trochanter pain syndrome. *Am J Sports Med* 2009; 37: 1981–90. 19439758 10.1177/0363546509334374

[5] Chandrasekaran S, Pavan Vemula S, Gui C et al. Clinical features that predict the need for operative intervention in gluteus medius tears. *Orthop J Sport Med* 2015; 3: 1–5. 10.1177/2325967115571079 PMC4555614 26535383

[6] Looney AM, Bodendorfer BM, Donaldson ST et al. Influence of fatty infiltration on hip abductor repair outcomes: a systematic review and meta-analysis. *Am J Sports Med* 2022; 50: 2568–80. 34495797 10.1177/03635465211027911

[7] Chandrasekaran S, Lodhia P, Gui C et al. Outcomes of open versus endoscopic repair of abductor muscle tears of the hip: a systematic review. *Arthrosc - J Arthrosc Relat Surg* 2015; 31: 2057–67.e2. 10.1016/j.arthro.2015.03.042 26033462

[8] Beck M, Leunig M, Ellis T et al. Advancement of the vastus lateralis muscle for the treatment of hip abductor discontinuity. *J Arthroplasty* 2004; 19: 476–80. 15188107 10.1016/j.arth.2003.11.014

[9] Fehm MN, Huddleston JI, Burke DW et al. Repair of a deficient abductor mechanism with Achilles tendon allograft after total hip replacement. *J Bone Jt Surg* 2010; 92: 2305–11. 10.2106/JBJS.I.01011 20926725

[10] Whiteside LA . Surgical technique: transfer of the anterior portion of the gluteus maximus muscle for abductor deficiency of the hip. *Clin Orthop Relat Res* 2012; 470: 503–10. 21796476 10.1007/s11999-011-1975-y PMC3254750

[11] Bogunovic L, Lee SX, Haro MS et al. Application of the Goutallier/Fuchs rotator cuff classification to the evaluation of hip abductor tendon tears and the clinical correlation with outcome after repair. *Arthrosc - J Arthrosc Relat Surg* 2015; 31: 2145–51. 10.1016/j.arthro.2015.04.101 26188781

[12] Kovalenko B, Bremjit P, Fernando N. Classifications in brief: Tönnis classification of hip osteoarthritis. *Clin Orthop Relat Res* 2018; 476: 1680–4. 30020152 10.1097/01.blo.0000534679.75870.5f PMC6259761

[13] Crossley KM, Bennell KL, Cowan SM et al. Analysis of outcome measures for persons with patellofemoral pain: which are reliable and valid? *Arch Phys Med Rehabil* 2004; 85: 815–22. 15129407 10.1016/s0003-9993(03)00613-0

[14] Norman GR, Sloan JA, Wyrwich KW. Interpretation of changes in health-related quality of life: the remarkable universality of half a standard deviation. *Med Care* 2003; 41: 582–92. 12719681 10.1097/01.MLR.0000062554.74615.4C

[15] Alpaugh K, Chilelli BJ, Xu S et al. Outcomes after primaryopen or endoscopic abductor tendon repair in the hip: a systematic review of the literature. *Arthrosc J Arthrosc Relat Surg* 2015; 31:530–40. 10.1016/j.arthro.2014.09.001 25442666

[16] Makridis KG, Lequesne M, Bard H et al. Clinical and MRI results in 67 patients operated for gluteus medius and minimus tendon tears with a median follow-up of 4.6 years. *Orthop Traumatol Surg Res* 2014; 100: 849–53. 25453914 10.1016/j.otsr.2014.08.004

[17] Browning RB, Clapp IM, Alter TD et al. Superior gluteal reconstruction results in promising outcomes for massive abductor tendon tears. *Arthrosc Sport Med Rehabil* 2021; 3: e1321–7. 10.1016/j.asmr.2021.05.013 PMC8527271 34712970

[18] Maldonado DR, Annin S, Chen JW et al. Combined transfer of the gluteus maximus and tensor fasciae latae for irreparable gluteus medius tear using contemporary techniques. *JBJS Open Access* 2020; 5: e20.00085. 10.2106/JBJS.OA.20.00085 PMC7757833 33376925

[19] Balazs GC, Dooley M, Wang D et al. Gluteus maximus transfer for irreparable hip abductor tendon tears: technique and clinical outcomes. *Tech Orthop* 2021; 36: 87–91.

[20] Christofilopoulos P, Kenanidis E, Bartolone P et al. Gluteus maximus tendon transfer for chronic abductor insufficiency: the Geneva technique. *HIP Int* 2021; 31: 751–8. 32397754 10.1177/1120700020924330

[21] Chandrasekaran S, Darwish N, Pavan Vemula S et al. Outcomes of gluteus maximus and tensor fascia lata transfer for primary deficiency of the abductors of the hip. *HIP Int* 2017; 27: 567–72. 28605003 10.5301/hipint.5000504

